# Abstract of Meteorological Observations for July, 1854, Made at Philadelphia, Pa.

**Published:** 1854-09

**Authors:** James A. Kirkpatrick


*Abstract of Meteorological Observations for July,* 1854, *made at Philadelphia, Pa. Latitude* 39° 57′ 28″ *N., Longitude* 75° 10′ 40″ *W. from Greenwich.* By Prof. James A. Kirkpatrick.

| 1854. July. | Barometer. Daily Mean | Barometer. Mean Daily Range. | Thermom. Daily Mean | Thermom. Mean Daily Range | Dew Point 2 P.M. | Rain. | Prevailing Winds. | General Remarks. |
|---|---|---|---|---|---|---|---|---|
| | Inches. | Inches. | Deg. | Deg. | Deg. | Inch. | Points. | |
| 1 | 29.925 | .197 | 74.5 | 1.3 | 52.7 | | SW. | M. and aft. clear; ev. cloudy. |
| 2 | 30.020 | .094 | 77.0 | 1.7 | 47.7 | | SW. | Clear. |
| 3 | 29.919 | .100 | 80.7 | 4.5 | 62.3 | | SW. | Clear. |
| 4 | 29.820 | .099 | 84.3 | 3.3 | 51.3 | | (Var.) | Clear. |
| 5 | 29.783 | .056 | 87.0 | 5.2 | 64.7 | 0.011 | E. | Cloudy. *Barom. lowest* 29.711 in. |
| 6 | 29.932 | .153 | 82.5 | 5.0 | 59.0 | | (Var.) | M. and aft. cloudy; ev. clear. |
| 7 | 29.982 | .050 | 79.0 | 2.3 | 61.0 | | S. | Cloudy. |
| 8 | 29.965 | .032 | 82.0 | 3.3 | 67.0 | | SW. | M. cloudy; aft. and evening clear. |
| 9 | 29.866 | .099 | 83.0 | 2.0 | 69.0 | 1.032 | NE. | Cloudy. |
| 10 | 29.872 | .042 | 74.0 | 8.5 | 57.0 | | E. | Cloudy. |
| 11 | 29.914 | .057 | 73.5 | 3.2 | 50.7 | | NE. | M. and aft. cloudy; ev. clear. |
| 12 | 29.923 | .072 | 71.0 | 1.7 | 57.0 | | NE. | M. and aft. cloudy; ev. clear. |
| 13 | 30.021 | .098 | 69.0 | 3.0 | 54.0 | 0.870 | NE. | Cloudy. *Therm. lowest* 63°. |
| 14 | 29.950 | .071 | 69.5 | 0.5 | 67.0 | 0.092 | ENE. | Cloudy. |
| 15 | 29.940 | .018 | 71.5 | 3.3 | 65.3 | | ENE. | M. and aft. cloudy; ev. clear. |
| 16 | 29.986 | .046 | 75.0 | 2.8 | 57.7 | | (Var.) | Cloudy. |
| 17 | 29.983 | .042 | 74.0 | 2.7 | 55.0 | | N. | M. cloudy; aft. and ev. clear. |
| 18 | 29.895 | .088 | 80.0 | 6.2 | 53.7 | | (Var.) | Clear. |
| 19 | 29.861 | .034 | 81.0 | 1.8 | 51.3 | | NW. | Clear. |
| 20 | 29.812 | .048 | 87.0 | 4.7 | 56.3 | | (Var.) | Clear. |
| 21 | 29.846 | .034 | 90.5 | 2.3 | 54.7 | | (Var.) | Clear. *Therm. highest,* 100½. |
| 22 | 29.931 | .085 | 89.7 | 3.2 | 63.8 | | (Var.) | Clear. |
| 23 | 29.967 | .068 | 85.5 | 4.0 | 61.7 | 0.230 | SSW. | Clear. |
| 24 | 29.939 | .047 | 79.0 | 4.7 | 70.3 | 1.382 | (Var.) | Cloudy. |
| 25 | 29.873 | .066 | 81.5 | 4.8 | 69.5 | 0.076 | (Var.) | Cloudy. |
| 26 | 29.826 | .047 | 83.0 | 2.7 | 71.3 | | SW. | Cloudy. |
| 27 | 30.016 | .190 | 76.5 | 7.3 | 52.0 | | NW. | Clear. |
| 28 | 30.079 | .098 | 77.0 | 2.7 | 52.3 | | (Var.) | Clear. *Barom. highest* 30.118 in. |
| 29 | 29.946 | .133 | 76.5 | 3.7 | 72.0 | 0.144 | SW. | Cloudy. |
| 30 | 29.901 | .086 | 81.0 | 4.3 | 55.3 | | (Var.) | Clear |
| 31 | 30.008 | .107 | 76.5 | 3.3 | 53.3 | | (Var.) | M. clear; aft. and ev. cloudy. |
| Means for July. 1854 | 29.926 | .079 | 79.1 | 3.6 | 59.2 | 3.837 | N. 71° W. 41—100. | |
| 1853 | 29.955 | | 75.1 | | 57.6 | 8.630 | | |
| 1852 | 29.903 | | 76.9 | | 55.9 | 4.060 | | |
| 3years | 29.928 | | 77.0 | | 57.6 | 5.509 | S. 82° W. 24—100. | |

The extreme range of the barometer during the month was 0.407 of an inch, and of the thermometer 37½°.

